# Supplementary material for: CombPDX: a unified statistical framework for evaluating drug synergism in patient-derived xenografts
Source: Sci Rep. 2022 Jul 29;12:12984. doi: 10.1038/s41598-022-16933-6 (PMC9338066; doi:10.1038/s41598-022-16933-6)
Supplement: Supplementary file 2 — Supplementary Information 2. [file 41598_2022_16933_MOESM2_ESM.docx]

**Supplementary Material**

Table of Contents

[Section S1: Simulation 1](#_Toc98850461)

[Section S2: Statistical Analysis 2](#_Toc98850462)

[Section S3: Method 3](#_Toc98850463)

[Section S3.1 Tumor Volume Data Processing 3](#_Toc98850464)

[Section S3.2: Treatment Effect 4](#_Toc98850465)

[Section S3.3: Highest Single Agent (HSA) 5](#_Toc98850466)

[Section S3.4: Response Additive (RA) 7](#_Toc98850467)

[Section S3.5: Bliss Independence (BI) 8](#_Toc98850468)

[Section S3.6: Comparison of three method 9](#_Toc98850469)

[Section S4: Tutorial 11](#_Toc98850470)

[Section S5: Supplementary Figures 13](#_Toc98850471)

[Section S6: Supplementary Tables 19](#_Toc98850472)

# **Section S1: Simulation**

To calculate the coverage probability, we generated data from the following steps:

1. The expected tumor volumes for the control group groups and single-agent groups were generated from the following differential equations (Winsor 1932; Ribba et al. 2014).
   Control group:

$$\frac{d\mu_{C}(t)}{dt}=-\alpha\mu_{C}\left( t \right)\log\left( \mu_{C}\left( t \right)-\mu_{C}\left( 0 \right)\exp\left( \frac{A}{\alpha} \right) \right)$$

Single-agent treatment group:

$$\frac{d\mu_{g}\left( t \right)}{dt}=-\alpha\mu_{g}\left( t \right)\log\left( \mu_{g}\left( t \right)-\mu_{g}\left( 0 \right)\exp\left( \frac{A}{\alpha} \right) \right)-\beta_{g}t\mu_{g}\left( t \right)exp\left( -k_{g}t \right)$$

where $\mu_{g}(t)$ is the tumor volume at time *t*, $\mu_{g}\left( 0 \right)$ is the log tumor volume at time 0. Without loss of generality, we set $\mu_{g}\left( 0 \right)$ to be 1.

1. We generated the expected tumor volumes for combination drug groups under each of the reference model:
   1. Highest Single Agent: $\mu_{AB}^{\left( HSA \right)}\left( t \right)=min\left( \mu_{A}\left( t \right),\mu_{B}\left( t \right) \right)$,
   2. Response Additive: $\mu_{AB}^{\left( RA \right)}\left( t \right)= \mu_{A}\left( t \right) + \mu_{B}\left( t \right) - \mu_{C}\left( t \right)$,
   3. Bliss Independence: $\mu_{AB}^{\left( BI \right)}\left( t \right) =\mu_{A}\left( t \right) \mu_{B}\left( t \right) /\mu_{C}\left( t \right)$,
2. The observed tumor volumes were generated from $\nu_{g}\left( t \right)=\mu_{g}\left( t \right)\times\epsilon$*,* where $\epsilon\sim logNormal\left( 0, 0.2 \right)$
3. We used the two scenarios:
   1. Model 1: $\left( A,\alpha,\beta_{A}, k_{A},\beta_{B},k_{b} \right)=$ (0.03, 0.01, 0.0064, 0.08, 0.015, 0.1) and
   2. Model 2:$\left( A,\alpha,\beta_{A}, k_{A},\beta_{B},k_{b} \right)=$ (0.02, 0.01, 0.018, 0.12, 0.01, 0.1).

The expected tumor growth curves for these simulation settings are shown in Figure S1. We used the mean tumor volume at day 21 as the response metrics. For each scenario, we generated 1000 replications with sample size N to be 5, or 10 in each group. The bootstrap interval was based on 1000 replications.

# **Section S2: Statistical Analysis**

Criteria for selecting a combination therapy experiment in a PDX model were as follows: 1) The control group, combination treatment group, and its individual single agent groups are available; and 2) each treatment has at least three mice with each mouse having at least three tumor measurements. Analyses were performed for each PDX model and each combination of drugs if the corresponding monotherapies exist. For each four-armed model, we evaluated the combination index of combination therapy using relative tumor volume. We assessed the combination effect using relative tumor volume at each day for up to day 21 days (or the last day in which each treatment group has at least three samples). Analyses were conducted under three definitions of drugs’ independence.

In pathway association analysis, pathways with less than 10 genes were filtered out, resulting 5164 in total. The PDX models were profiled for their gene expression using RNA-seq with 15,732 genes after filtering out genes whose 75% percentile was less than 20. In addition, criteria for selecting a PDX model were as follows: 1) RNA-Seq data is available and 2) the effect of its monotherapies fulfill the BI assumption (i.e 0 $\leq\delta_{A},\delta_{B}\leq1$).

All analyses were performed in R v4.0.2.

# **Section S3: Method**

In a fixed-dose two-drug combination PDX experiment, tumor-bearing mice are randomly assigned to one of the four treatment arms: control group (denoted as C), two single-drug treatment groups (denoted as A or B), and combination treatment group (denoted as AB) at the baseline. The combPDX requires a long data matrix as an input where each of the tumor volume measures are stacked by rows with four columns: mice ID, treatment, day, and tumor volume. Table S4 presents the description of these metrics and Table S5 shows an example of input data.

## **Section S3.1 Tumor Volume Data Processing**

Due to the variation of initial tumor volumes across mice, the response for an individual animal at each time point is defined by the relative tumor volume, which is the raw tumor volume divided by the initial tumor volume of the mouse. We denote $v_{t}$ as the relative tumor volume for a mouse at time$t$. For subjects where there is no tumor volume measurement at time $t$, but which have flanking volume measurements at time $t_{0}$ and $t_{1}$, we use linear interpolation to impute the relative tumor volume at time *t*:

$v_{t} = v_{t_{0}} +\frac{v_{t_{1}}-v_{t_{0}}}{t_{1}-t_{0}} (t - t_{0})$.

Denote that response for mouse $i$*,* $v_{i}$, follows an independent identical distribution with mean and variance to be $\mu_{g}, \sigma_{g}^{2}$ respectively, where $g=C,A, B, AB$ represents control, drug A or B and combination group respectively. By Central limit theorem,$\bar{v}_{g}\sim N\left( \mu_{g}, \frac{\sigma_{g}^{2}}{n_{g}} \right)$ with a sufficiently large sample size. Denote that $\mu=\left( \mu_{c}, \mu_{A},\mu_{B},\mu_{AB} \right)$ and its estimators $\bar{v}=\left( \bar{v}_{c}, \bar{v}_{A},\bar{v}_{B},\bar{v}_{AB} \right)$ with $cov\left( \bar{v} \right)=diag\left( \frac{\sigma_{C}^{2}}{n_{C}},\frac{\sigma_{A}^{2}}{n_{A}},\frac{\sigma_{B}^{2}}{n_{B}},\frac{\sigma_{AB}^{2}}{n_{AB}} \right)$ where *diag()* denotes the diagonal matrix. By Delta method, we have $\hat{CI}\sim N\left( CI, var\left( \hat{CI} \right)=\nabla{CI}^{T}cov\left( \bar{v} \right)\nabla CI \right)$, where $CI$ is a function of $\mu$, and $\nabla CI$ is the gradient of $CI$ with respect to $\mu$.

## **Section S3.2: Treatment Effect**

To access the combination effect of two drugs, we use an effect-based approach that directly compares the effect of the combination to the effects of its individual components. The effect $\delta_{g}$ is quantified by the mean reduction in the relative tumor volumes between treatment and control groups divided by the control mean.

|  | $\delta_{g}=\frac{\mu_{C}- \mu_{g}}{\mu_{C}} \mathrm{for}g\in A, B, AB$, |  |
| --- | --- | --- |

where $\mu_{g}$ is the mean of the relative tumor volumes for group $g$, and the relative tumor volume is the raw tumor volume divided by the initial tumor volume of the mouse to account for the individual variation. A large $\delta_{g}$ value indicates a strong treatment effect. While this has been widely used as the tumor growth inhibition with predetermined cutoffs of declaring an antitumor activity (Houghton et al. 2007; Mer et al. 2019; Ortmann et al. 2020),

The gradient with respect to $(\mu_{C}, \mu_{g})$ is

$$\Delta\delta_{g}=\left( \frac{\mu_{g}}{\mu_{C}^{2}} , - \frac{1}{\mu_{C}} \right)^{T}$$

Then the variance can be expressed as

$$var\left( \hat{\delta_{g}} \right)= \left( \frac{\mu_{g}^{2}}{\mu_{C}^{4}}\frac{\sigma_{C}^{2}}{n_{C}} , \frac{1}{\mu_{C}^{2}}\frac{\sigma_{g}^{2}}{n_{g}} \right)$$

The estimate of standard error is

$$\hat{se} \left( \hat{\delta_{g}} \right)= \left( \frac{\bar{v}_{g}^{2}}{\bar{v}_{C}^{4}}\frac{\hat{\sigma}_{C}^{2}}{n_{C}} , \frac{1}{\bar{v}_{C}^{2}}\frac{\hat{\sigma}_{g}^{2}}{n_{g}} \right)^{1/2}$$

where $\bar{v}_{g}$ is the mean response, $\hat{\sigma}_{g}^{2}=\sum_{i=1}^{n_{g}} \frac{{(v}_{gi}-\bar{v}_{g})^{2}}{n_{g}-1}$, and $n_{g}$ is the number of mice in the treatment $g$.

The lower bound of a one-sided *100(1-*$\alpha)\%$ confidence interval for a combination index can be calculated using the Delta method,

$$\hat{\delta}_{g}-z_{1-\alpha}\hat{se}\left( \hat{\delta}_{g} \right)$$

where$\hat{\delta}_{g}= \frac{\bar{v}_{C}- \bar{v}_{g}}{\bar{v}_{C}}$, ${\hat{se}\left( \hat{\delta}_{g} \right)= \left( \frac{\bar{v}_{g}^{2}}{\bar{v}_{C}^{4}}\frac{\hat{\sigma}_{C}^{2}}{n_{C}}+\frac{1}{\bar{v}_{C}^{2}}\frac{\hat{\sigma}_{g}^{2}}{n_{g}} \right)}^{1/2}$, $\bar{v}_{g}\mathrm{and}\hat{\sigma}_{g}^{2}$ are the sample mean and sample variance for $g\in C,A, B, AB$, respectively. $z_{1-\alpha}$ is the 100$\left( 1-\alpha\right)\%$^th^ quantile of standard normal distribution. The lower bound greater than 0 indicates that the treatment is more effective than the control group at the significance level of 0.05.

## **Section S3.3: Highest Single Agent (HSA)**

If combination drug $\mathrm{AB}$ is synergistic, then under Highest Single Agent (Berenbaum 1989; Lehár et al. 2007; Geary 2013; Foucquier and Guedj 2015)

$\frac{max\left( \delta_{A}, \delta_{B} \right)}{\delta_{AB}}<1$,

$$\Longleftrightarrow\frac{\max\left( \frac{\mu_{C}-\mu_{A}}{\mu_{C}}, \frac{\mu_{C}-\mu_{B}}{\mu_{C}} \right)}{\frac{\mu_{C}-\mu_{AB}}{\mu_{C}}}<1$$

$$\Longleftrightarrow\frac{\min\left( \mu_{A}, \mu_{B} \right)}{\mu_{AB}}>1$$

which is equivalent to test the combination index

|  | $CI = log(\mu_{g}) - log(\mu_{AB})$*.* |  |
| --- | --- | --- |

where $g=A or B$ indicates the monotherapy having better treatment effect. We choose the single agent $g$ having larger $\delta$ in equation (1), or narrower confidence interval when the single agent effects are equal. $CI <0, =0, >0$represent antagonistic, independent, and synergistic combination effects respectively. The estimates of combination index is calculated as

$\hat{CI}= log(\bar{v}_{g}) - log(\bar{v}_{AB})$*,*

where $\bar{v}_{g}$ is the mean response for treatment $g$*.*

The gradient of $CI$ with respect to $\mu$ is

$$\nabla CI= \left\{ \begin{aligned} \left( 0, \frac{1}{\mu_{A}}, 0 , \right.\left. \frac{1}{\mu_{AB}} \right)^{T} if g=A \\ \left( 0,0, \frac{1}{\mu_{B}}, \right.\left. \frac{1}{\mu_{AB}} \right)^{T} if g=B \end{aligned} \right.$$

Then the standard error is

$$se\left( \hat{CI} \right)=\left( \frac{1}{\mu_{g}^{2}}\frac{\sigma_{g}^{2}}{n_{g}} + \frac{1}{\mu_{AB}^{2}}\frac{\sigma_{AB}^{2}}{n_{AB}} \right)^{1/2}$$

Substitute $\mu_{g} \mathrm{and}\sigma_{g}$ with their estimators, we have the estimated standard error:

$$\hat{se}(\hat{CI}) \simeq\left( \frac{1}{\bar{v}_{g}^{2}}\frac{\hat{\sigma}_{g}^{2}}{n_{g}} + \frac{1}{\bar{v}_{AB}^{2}}\frac{\hat{\sigma}_{AB}^{2}}{n_{AB}} \right)^{1/2}$$

where $\hat{\sigma}_{g}^{2}=\sum_{i=1}^{n_{g}} \frac{{(v}_{gi}-\bar{v}_{g})^{2}}{n_{g}-1}$, and $n_{g}$ is the number of mice in the treatment $g$ group. A two-sided $100(1-\alpha)\%$ confidence interval is $\left( \hat{CI}-z_{1-\alpha/2}\hat{se}\left( \hat{CI} \right), \hat{CI}+z_{1-\alpha/2}\hat{se}\left( \hat{CI} \right) \right)$, where $z_{1-\alpha}$ is the ($1-\alpha$) percentile of the standard normal distribution.

## **Section S3.4: Response Additive (RA)**

A combination drug is considered synergistic under response additive (Slinker 1998) (Foucquier and Guedj 2015) if it shows an enhanced effect than the summation of the effects of the two monotherapies. It can be represented as

$\frac{\delta_{A}+ \delta_{B}}{\delta_{AB}}<1$*,*

$$\Longleftrightarrow\frac{\frac{\mu_{C}-\mu_{A}}{\mu_{C}}+ \frac{\mu_{C}-\mu_{B}}{\mu_{C}}}{\frac{\mu_{C}-\mu_{AB}}{\mu_{C}}}<1$$

$$\Longleftrightarrow\frac{\mu_{A}+ \mu_{B}}{\mu_{C}+\mu_{AB}}>1$$

which is equivalent to assess the combination index

$CI=log\left( \mu_{A}+\mu_{B} \right)-log\left( \mu_{AB}+ \mu_{C} \right)$*,*

where$CI<0, =0, >0$ represent antagonistic, independent, and synergistic combination effects, respectively. The estimates of combination index is

$\hat{CI}= log(\bar{v}_{A}+\bar{v}_{B}) - log(\bar{v}_{AB}+\bar{v}_{C})$*.*

The gradient of $\mathrm{CI}$ with respect to $\mu$ is

$$\nabla CI= \left( \frac{1}{(\mu_{C}+\mu_{AB})},\frac{1}{{(\mu}_{A}+\mu_{B})}, \frac{1}{{(\mu}_{A}+\mu_{B})} , \right.\left. \frac{1}{(\mu_{C}+\mu_{AB})} \right)^{T}$$

Then the variance is

$$var\left( \hat{CI} \right)=\frac{1}{{(\mu}_{A}+\mu_{B})^{2}}\frac{\sigma_{A}^{2}}{n_{A}}+\frac{1}{{(\mu}_{A}+\mu_{B})^{2}}\frac{\sigma_{B}^{2}}{n_{B}} +\frac{1}{(\mu_{C}+\mu_{AB})^{2}}\frac{\sigma_{C}^{2}}{n_{C}}+\frac{1}{(\mu_{C}+\mu_{AB})^{2}}\frac{\sigma_{AB}^{2}}{n_{AB}}$$

Substitute $\mu_{A} \mathrm{and}\sigma_{g}$ with their estimators, we have the estimated standard error:

$\hat{se}(\hat{CI}) \simeq\left( \frac{1}{{(\bar{v}}_{A}+\bar{v}_{B})^{2}}\frac{\hat{\sigma}_{A}^{2}}{n_{A}}+\frac{1}{{(\bar{v}}_{A}+\bar{v}_{B})^{2}}\frac{\hat{\sigma}_{B}^{2}}{n_{B}} +\frac{1}{(\bar{v}_{C}+\bar{v}_{AB})^{2}}\frac{\hat{\sigma}_{C}^{2}}{n_{C}}+\frac{1}{(\bar{v}_{C}+\bar{v}_{AB})^{2}}\frac{\hat{\sigma}_{AB}^{2}}{n_{AB}} \right)^{1/2}$

where $\bar{v}_{g}$ is the mean response, $\hat{\sigma}_{g}^{2}=\sum_{i=1}^{n_{g}} \frac{{(v}_{gi}-\bar{v}_{g})^{2}}{n_{g}-1}$, and $n_{g}$ is the number of mice in the treatment $g$. A two-sided $100(1-\alpha)\%$ confidence interval for the combination index is,

$\left( \hat{CI}-z_{1-\alpha/2}\hat{se}\left( \hat{CI} \right), \hat{CI}+z_{1-\alpha/2}\hat{se}\left( \hat{CI} \right) \right)$,

where $z_{1-\alpha}$ is the ($1-\alpha$) percentile of the standard normal distribution.

## **Section S3.5: Bliss Independence (BI)**

The Bliss Independence (Bliss 1939; Berenbaum 1989; Greco et al. 1995; Geary 2013; Foucquier and Guedj 2015) considers the effect of a treatment as probability, so it assumes that $\delta_{g}$ ranges from 0 to 1. Under probabilistic independence, we expect that $\frac{\delta_{A} + \delta_{B} -\delta_{A}\delta_{B}}{\delta_{AB}}=1$. If the combined drug is synergistic under the Bliss Independence, then we have

$\frac{\delta_{A} + \delta_{B} - \delta_{A}\delta_{B}}{\delta_{AB}}<1$*.*

$$\Longleftrightarrow\frac{\mu_{C}-\mu_{AB}}{\mu_{C}}>\frac{\mu_{C}-\mu_{A}}{\mu_{C}}+ \frac{\mu_{C}-\mu_{B}}{\mu_{C}}-\left( \frac{\mu_{C}-\mu_{A}}{\mu_{C}} \right)\left( \frac{\mu_{C}-\mu_{B}}{\mu_{C}} \right)$$

$$\Longleftrightarrow\frac{\mu_{C}-\mu_{A} +\mu_{C}-\mu_{B} -\mu_{C}+ \mu_{A}+ \mu_{B}-\frac{\mu_{A}\mu_{B}}{\mu_{C}}}{\mu_{C}-\mu_{AB}}<1$$

$$\Longleftrightarrow\frac{\mu_{A}\mu_{B}}{\mu_{AB}\mu_{C}}>1$$

It is equivalent to define the combination index as

$CI=log(\mu_{A}) +log(\mu_{B})-log(\mu_{C})-log(\mu_{AB})$*,*

where $CI <0, =0, >0$ represent antagonistic, independent and synergistic combination effects respectively. The estimates of combination index is

$\hat{CI}=log(\bar{v}_{A}) +log(\bar{v}_{B})-log(\bar{v}_{C}) - log(\bar{v}_{AB})$*.*

The gradient of $CI$ with respect to $\mu$ is

$$\nabla CI= \left( \frac{1}{\mu_{C}},\frac{1}{\mu_{A}}, \frac{1}{\mu_{B}}, \right.\left. \frac{1}{\mu_{AB}} \right)^{T}$$

Then the variance is

$$var\left( \hat{CI} \right)=\frac{1}{\mu_{C}^{2}}\frac{\sigma_{A}^{2}}{n_{A}}+\frac{1}{\mu_{A}^{2}}\frac{\sigma_{B}^{2}}{n_{B}} +\frac{1}{\mu_{B}^{2}}\frac{\sigma_{C}^{2}}{n_{C}}+\frac{1}{\mu_{AB}^{2}}\frac{\sigma_{AB}^{2}}{n_{AB}}$$

Substitute $\mu_{A} \mathrm{and}\sigma_{g}$ with their estimators, we have the estimated standard error:

$\hat{se}(\hat{CI}) \simeq\left( \frac{1}{\bar{v}_{A}^{2}}\frac{\hat{\sigma}_{A}^{2}}{n_{A}}+\frac{1}{\bar{v}_{B}^{2}}\frac{\hat{\sigma}_{B}^{2}}{n_{B}} +\frac{1}{\bar{v}_{C}^{2}}\frac{\hat{\sigma}_{C}^{2}}{n_{C}}+\frac{1}{\bar{v}_{AB}^{2}}\frac{\hat{\sigma}_{AB}^{2}}{n_{AB}} \right)^{1/2}$

where $\bar{v}_{g}$ is the mean response, $\hat{\sigma}_{g}^{2}=\sum_{i=1}^{n_{g}} \frac{{(v}_{gi}-\bar{v}_{g})^{2}}{n_{g}-1}$, and $n_{g}$ is the number of mice in the treatment $g$ group. A two-sided $100(1-\alpha)\%$ confidence interval is $\left( \hat{CI}-z_{1-\alpha/2}\hat{se}\left( \hat{CI} \right), \hat{CI}+z_{1-\alpha/2}\hat{se}\left( \hat{CI} \right) \right)$, where $z_{1-\alpha}$ is the ($1-\alpha$) percentile of the standard normal distribution.

## **Section S3.6: Comparison of three method**

Let $\delta_{AB}^{(HSA)}, \delta_{AB}^{(RA)}, \delta_{AB}^{(BI)}$ denote the expected effect of the combination drug under each of the three reference models. To conclude synergy, the observed combination effect $\delta_{AB}$ needs to be larger than the reference $\delta_{AB}^{(\cdot)}$. In the following derivation, we expect single-agent treatments to be more effective than the control. Without loss of generality, let $\delta_{A}\geq\delta_{B}$, then we have

$\delta_{AB}^{(HSA)}=\max\left( \delta_{A}, \delta_{B} \right)=\delta_{A}$.

Since $0 {\leq\delta}_{g}\leq1$,

$${\delta_{AB}^{(HSA)}=\delta}_{A}\leq\delta_{A}+\delta_{B}\left( 1-\delta_{A} \right)\leq\delta_{A} + \delta_{B} - \delta_{A}\delta_{B}= \delta_{AB}^{(BI)}$$

Then we have

$$\delta_{AB}^{(BI)}=\delta_{A} + \delta_{B} - \delta_{A}\delta_{B}\leq\delta_{A} + \delta_{B}= \delta_{AB}^{(RA)}$$

So, in summary, we have three baselines in the following order:

$$\delta_{AB}^{(HSA)}\leq\delta_{AB}^{(BI)}\leq\delta_{AB}^{\left( RA \right)}$$

We show that HSA has the smallest threshold while RA has the highest.

Next, we compare the $CIs$ for the three reference models. Let $\mu_{0}^{HSA}$, $\mu_{0}^{RA}$ and $\mu_{0}^{BI}$ be the expected tumor volume in combination group when its corresponding $CI_{\left( \cdot\right)}=0$. If the $\mu_{AB}$ smaller than the $\mu_{0}^{\left( \cdot\right)}$, synergistic effect is detected under such reference model. Solving the equations $CI_{HSA}=0$, $CI_{RA}=0$, and $CI_{BI}=0$, we have $\mu_{0}^{HSA}=\mu_{A}$ , $\mu_{0}^{RA}=\mu_{A}+ \mu_{B}-\mu_{C}$, $\mu_{0}^{BI}=\mu_{A} \mu_{B}/\mu_{C}$. When $0\leq\delta_{A},\delta_{B}\leq1$, we have $\mu_{0}^{BI}\leq\mu_{0}^{RA}$ and

$$\mu_{0}^{RA}- \mu_{0}^{BI}= \mu_{A}+ \mu_{B}-\mu_{C}-\mu_{A}\frac{\mu_{B}}{\mu_{C}}$$

Dividing $\mu_{C}$, we have

$$\frac{\mu_{A}}{\mu_{C}}+ \frac{\mu_{B}}{\mu_{C}}-1-\frac{\mu_{A}}{\mu_{C}}\frac{\mu_{B}}{\mu_{C}}=\frac{\mu_{A}}{\mu_{C}}\left( 1-\frac{\mu_{B}}{\mu_{C}} \right)- \left( 1-\frac{\mu_{B}}{\mu_{C}} \right)= -\left( 1-\frac{\mu_{B}}{\mu_{C}} \right)\left( 1- \frac{\mu_{A}}{\mu_{C}} \right)=-\delta_{A}\delta_{B}\leq0$$

and then we have $\mu_{0}^{RA}\leq\mu_{0}^{BI}\leq\mu_{0}^{HSA}$. Figure S4 illustrates that the maximum tumor volumes having synergistic effect ($CI >0$) in each reference model, given the mean tumor volume growth curves of the control group and single-agent treatment groups.

We compare the three $CI$s for a given $\mu_{AB}$,

$$CI_{BI}-CI_{HSA}= log\left( \mu_{A} \right)+log\left( \mu_{B} \right)-log\left( \mu_{C} \right)-log\left( \mu_{AB} \right)-log\left( \mu_{A} \right)+ log\left( \mu_{AB} \right)= log\left( \mu_{B} \right)-log\left( \mu_{C} \right) < 0$$

So, we have $CI_{BI}\leq CI_{HSA}$. Let $h\left( \mu_{AB} \right)= CI_{RA}-CI_{BI}= log\left( \mu_{A}+\mu_{B} \right)-log\left( \mu_{AB}+ \mu_{C} \right)- log\left( \mu_{A} \right)-log\left( \mu_{B} \right)+log\left( \mu_{C} \right)+log\left( \mu_{AB} \right).$Then the derivative with respect to $\mu_{AB}$ is $h^{'}\left( \mu_{AB} \right)= -\frac{1}{\mu_{AB}+ \mu_{C}}+ \frac{1}{\mu_{AB}}>0$. $h\left( \mu_{AB} \right)$increases as $\mu_{AB}$ increases and at $\mu_{AB}= \mu_{0}^{RA}$, $0=CI_{RA}\leq CI_{BI}$, so when $\mu_{AB}\leq\mu_{0}^{RA}$, we have $0\leq CI_{RA}\leq CI_{BI}\leq CI_{HSA}$.

In summary, the HSA is the least stringent baseline to conclude synergy since the observed combination effect is compared to the relative more effective component. The RA is the most stringent since the observed combination need to be more effective than two drug effect added together. The BI provides a compromise between these two methods.

# **Section S4: Tutorial**

The combPDX currently includes five tabs: Upload Dataset, Visualize Results, Power calculation, Download Results and Batch Analysis. These allow the users to test treatment effect as well as combination effect, view output figures and tables, download result and run batch analysis.

First, The Upload Dataset tab is presented in Figure S5. Data input boxes are provided to input the long format of longitudinal tumor volume. The combPDX supports two file types: .txt or .csv. After input the data, user needs to specify the following five columns to proceed analysis: Experiment ID, Mice ID, Treatment, Tumor Volume, Date. Table S4 shows the description of these metrics and Table S5 shows the example of sample data. After upload data and specify column names, combPDX provides PDX model summary and data preview in the right panel (Figure S5).

Next, the Visualize Results tab is presented in Figure S6. User needs to select PDX model, and treatment information for the four-arm experiments. Then, combPDX makes inference both locally and globally for up to the specified maximum days. Bootstrap confidence interval is recommended for small sample size. Though, we adjust initial tumor volume to account for the baseline variation, user can turn off this option if needed. Minimum number of observations is the criteria to include a mouse if it has more observations than such value. To make inference on a time point, the sample size in each group needs to exceed the minimum sample size. After click ‘run combPDX’, it provides profile plot, treatment effect, and Combination Index for each baseline model. One-sided 95% confidence interval is provided in treatment analysis, and two-sided 95% confidence interval is provided in CI analysis.

Then, the users can utilize Downloaded Results tab to download a table or .Rdata file with result from combPDX. Additionally, the Batch Analysis tab is presented in Figure S7 The input is similar to Visualize Result tab. An addition input required for testing multiple combination drugs is the symbol that indicates such combination drug with the default to be “+”. After clicking the download bottom, a html report is generated including all the results.

Finally, the Power Calculation tab provides sample size calculation and power calculation for PDX models.

# **Section S5: Supplementary Figures**


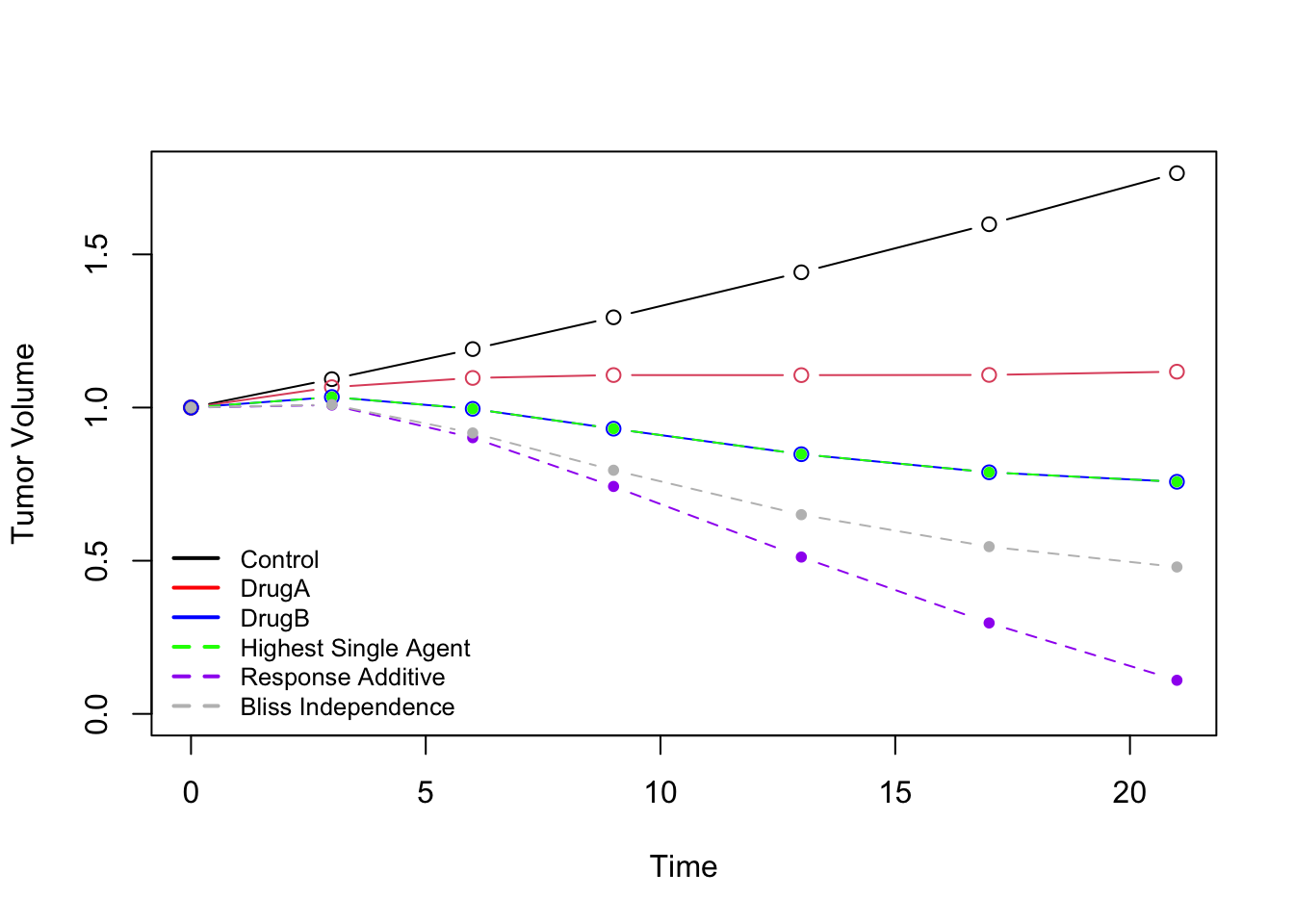

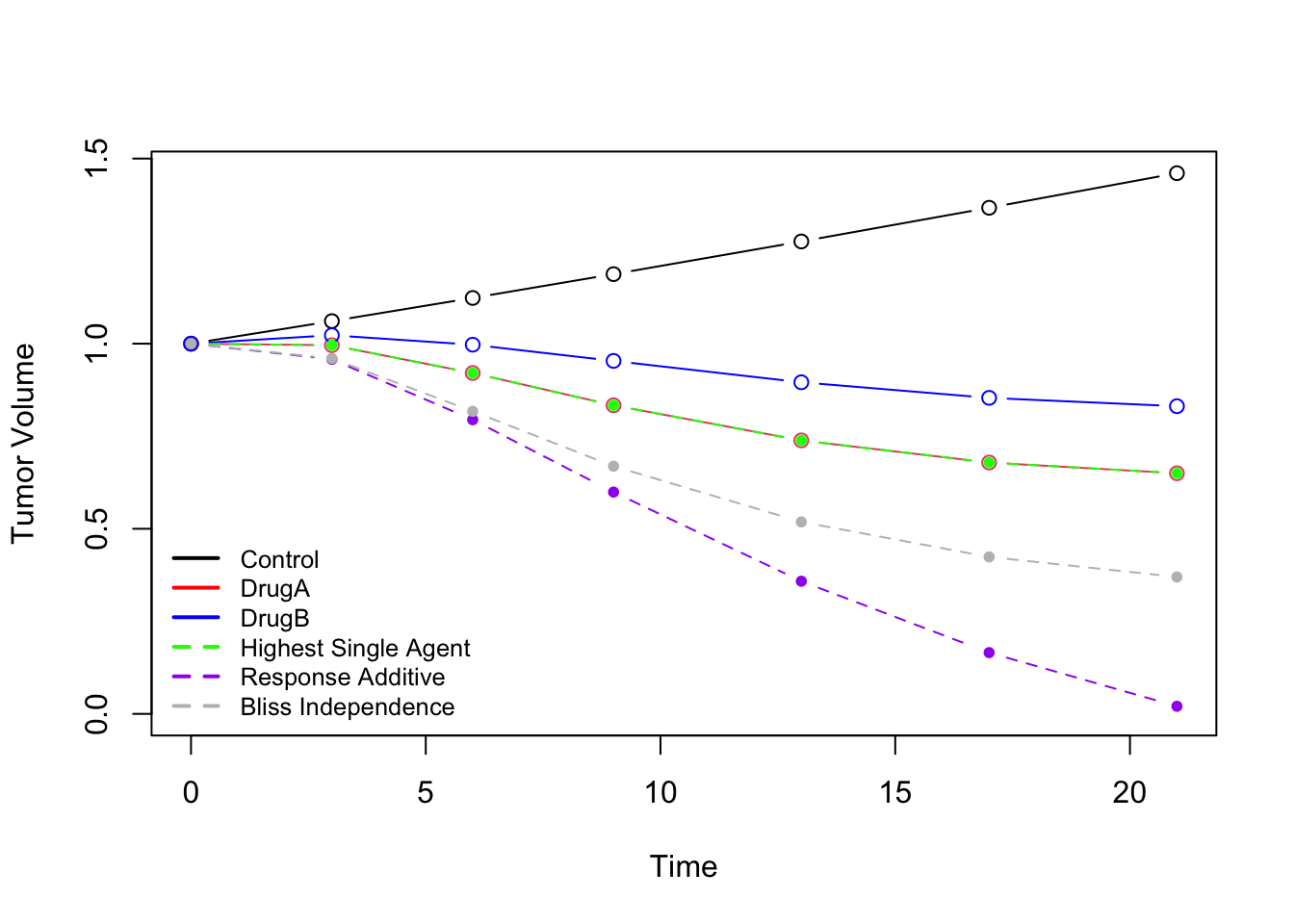


Figure S1: Simulation settings under two scenarios with the parameters $\left( A,\alpha,\beta_{A}, k_{A},\beta_{B},k_{b} \right)$ to be: left panel (Model 1) (0.03, 0.01, 0.0064, 0.08, 0.015, 0.1), and right panel (Model 2) (0.02, 0.01, 0.018, 0.12, 0.01, 0.1). The solid lines represent the mean tumor growth curves of control group and single-agent group, and the dashed lines represent the maximum tumor volumes for combination therapy AB ($\mu_{AB}$) having synergistic effect ($CI >0$) under each reference model.


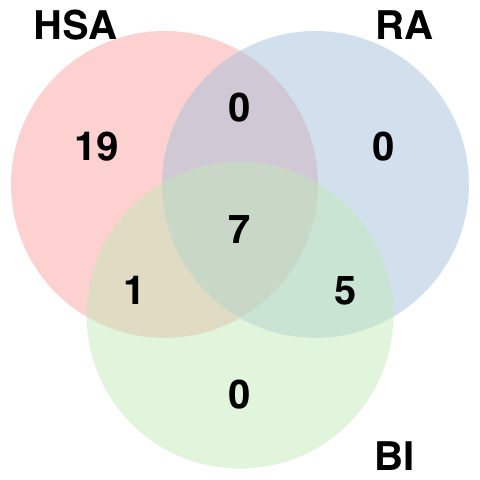

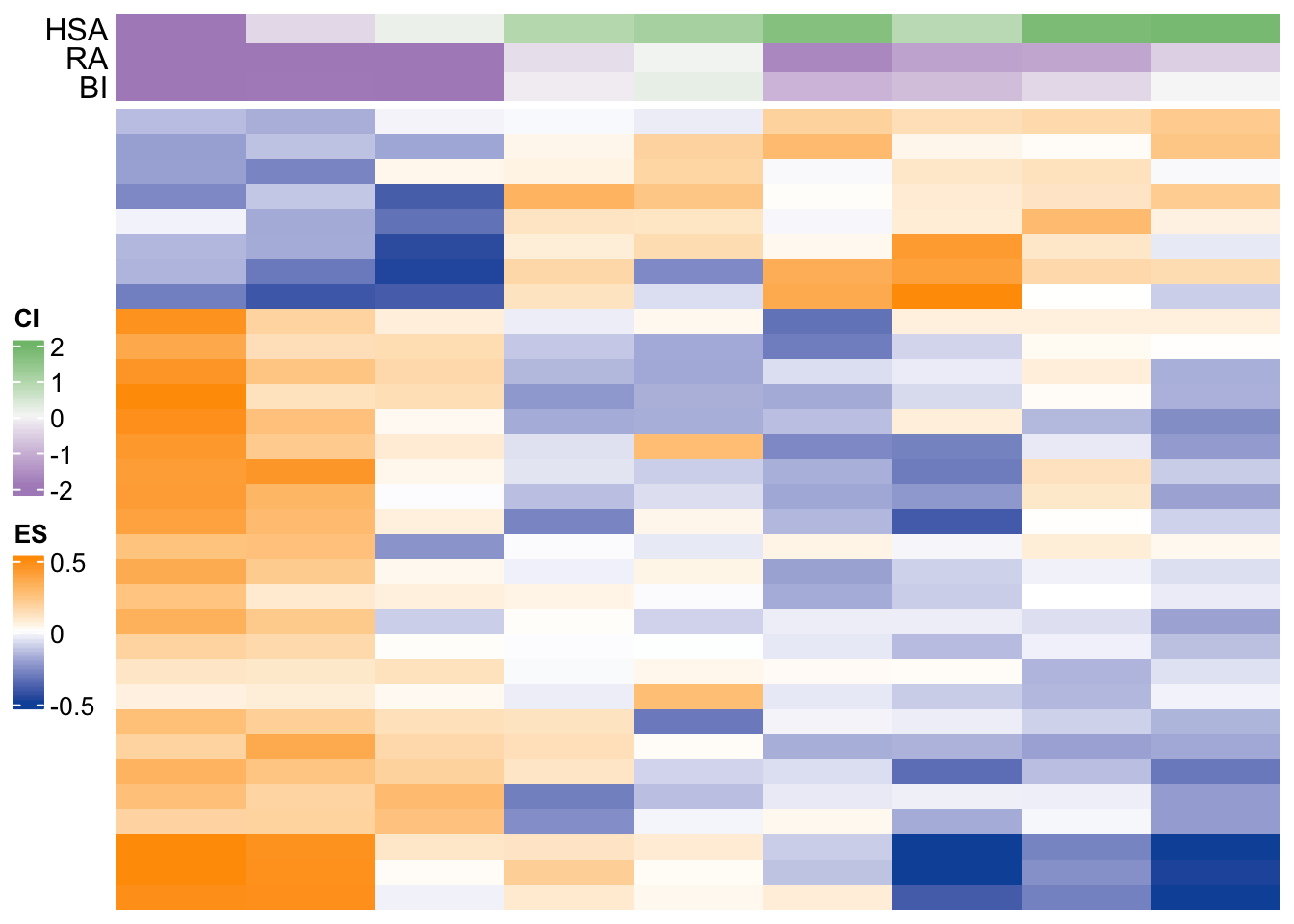


Figure S2: Differentially activated pathways at FDR of 0.1 for KRT232 plus Trametinib. (a) Venn diagram showing distribution of significantly expressed pathways. The figure illustrates the number of statistically significantly expressed pathways associated with each $gCI$. (b) Heatmap of baseline pathway enrichment score from GSVA with the row annotation to be $gCI$s. Each column represents a PDX model, and each row represents a gene whose enrichment score was significantly correlated with one or more $gCI$s using the distance correlation test.


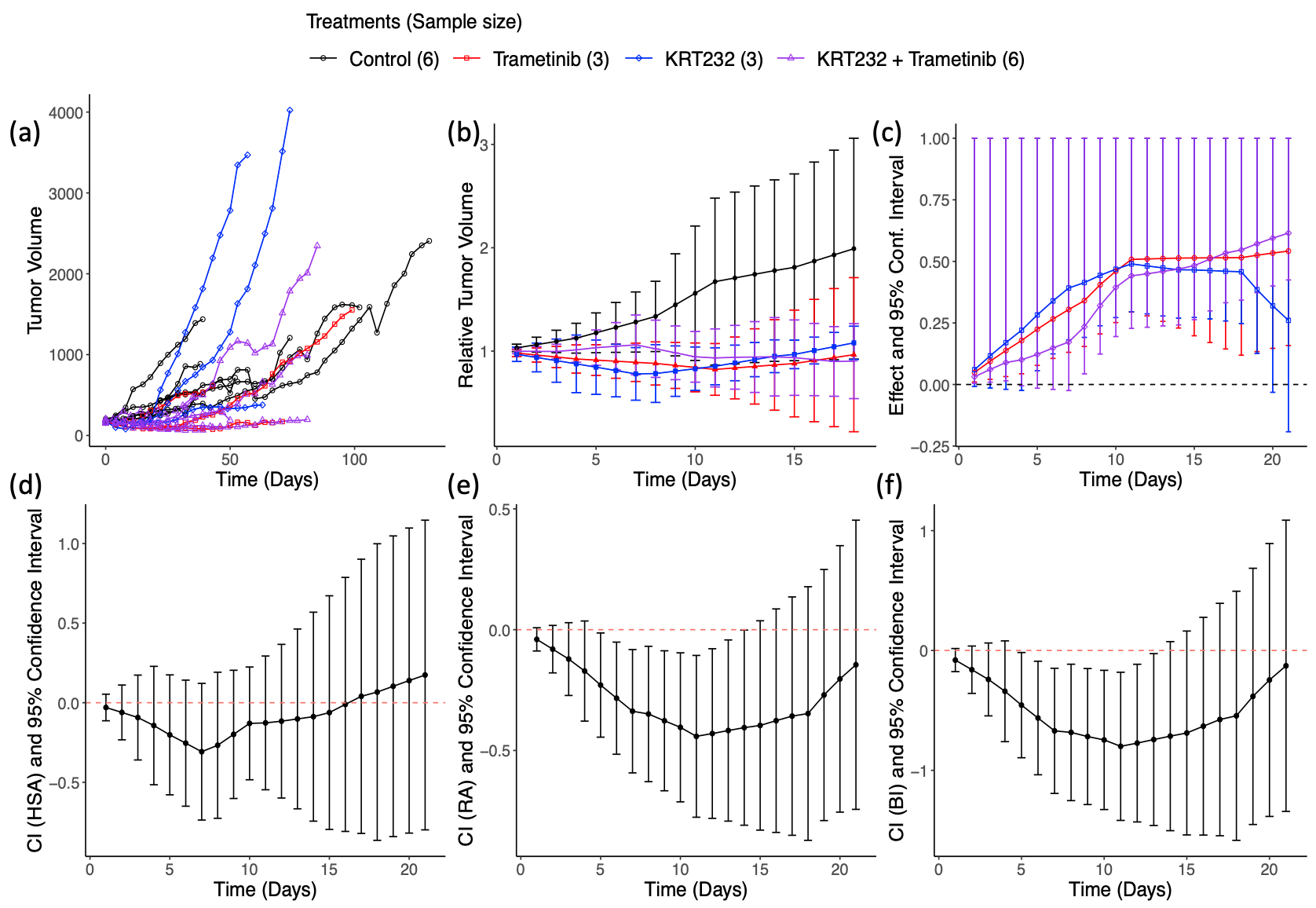


Figure S3: Effect and Combination Indices for KRT232 plus trametinib. (a) Profile plot of tumor volume. (b) Profile plot of relative tumor volume. Y-axis shows the mean $\pm$ standard error of relative tumor volume within each treatment group. (c) The drug effect for each treatment group relative to control group is quantified by tumor growth inhibition (TGI). The vertical line indicates the one-sided 95% confidence interval. (d)-(f) The joint action of combination drug under each reference model (HSA, RA, and BI) is assessed using a combination index. The vertical line indicates the two-sided 95% confidence interval.


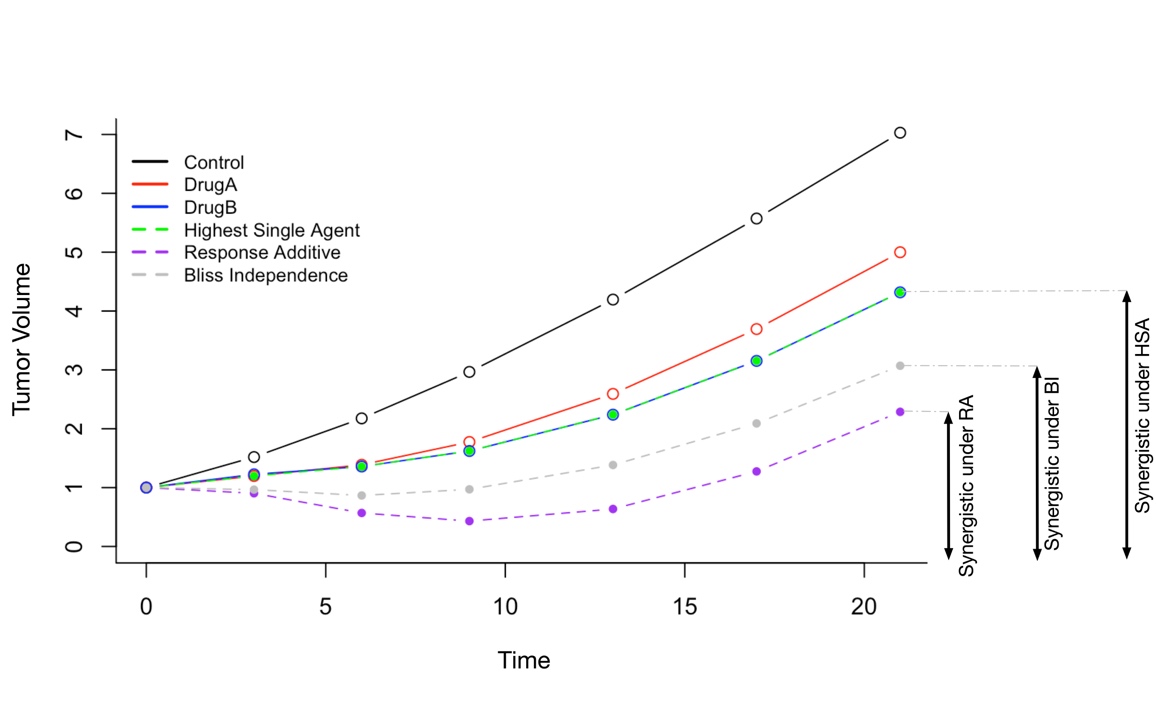


Figure S4: Illustration and comparison of three methods. The solid lines represent the mean tumor growth curves for control ($\mu_{C}$) and monotherapies A and B ($\mu_{A} \mathrm{and}$ $\mu_{B}$*),* and the dashed lines represent the maximum tumor volumes for combination therapy AB ($\mu_{AB}$) having synergistic effect ($CI >0$) under each reference model.


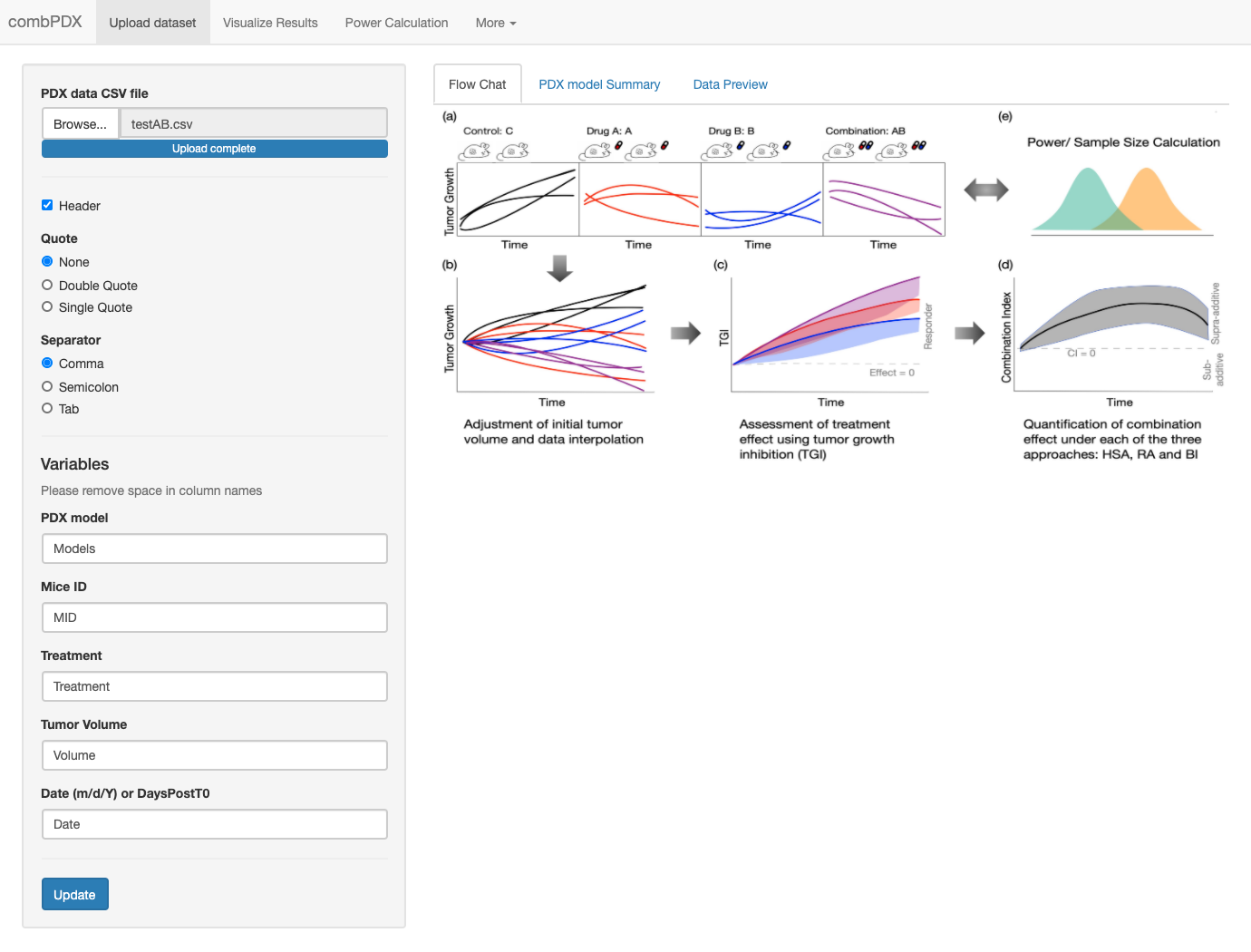


Figure S5: Job setup tab in combPDX includes data uploading, model summary and data preview panels.


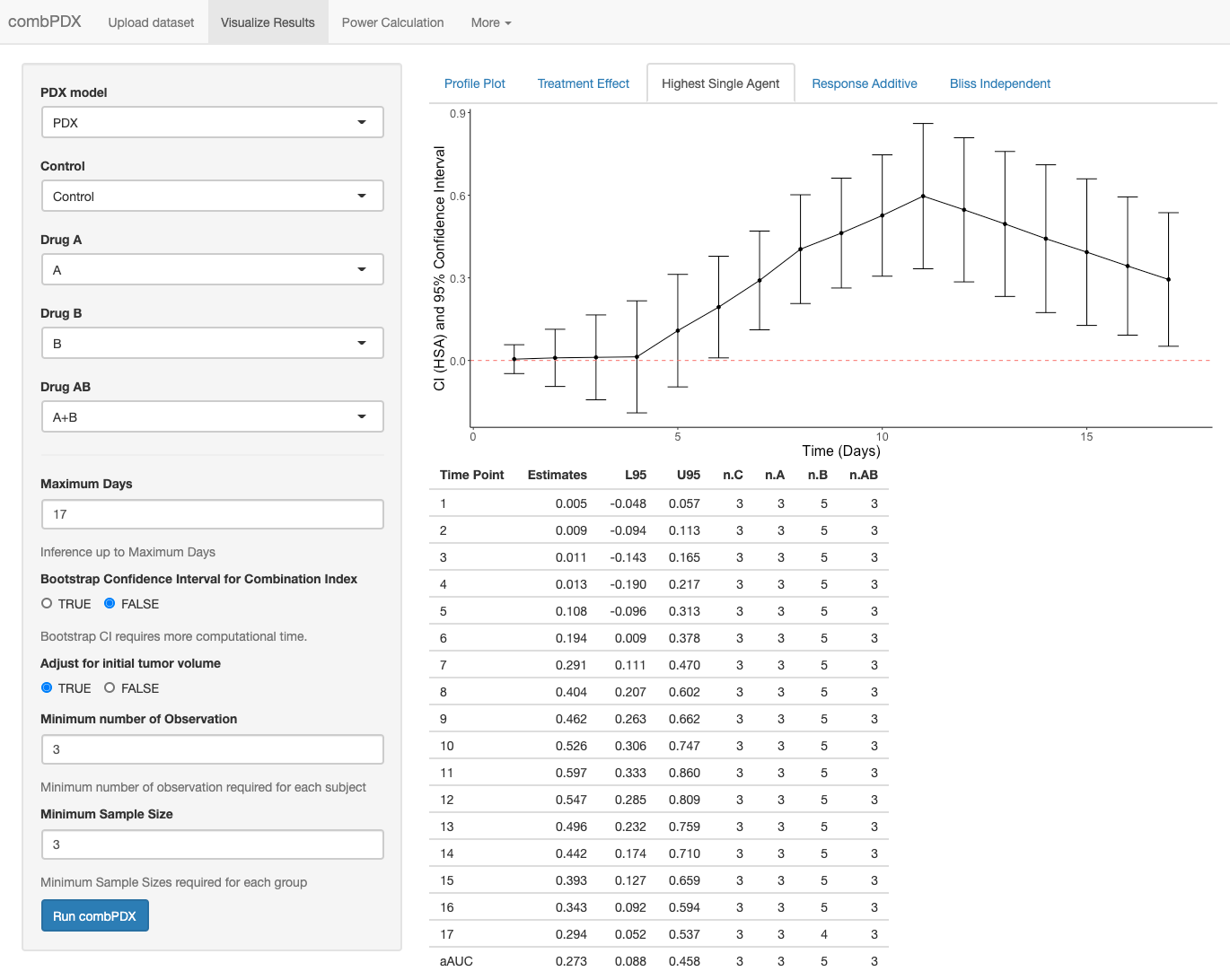


Figure S6: Result visualization tab in combPDX can present the profile plot of the raw data, treatment effect analysis, and Combination Index for three models.


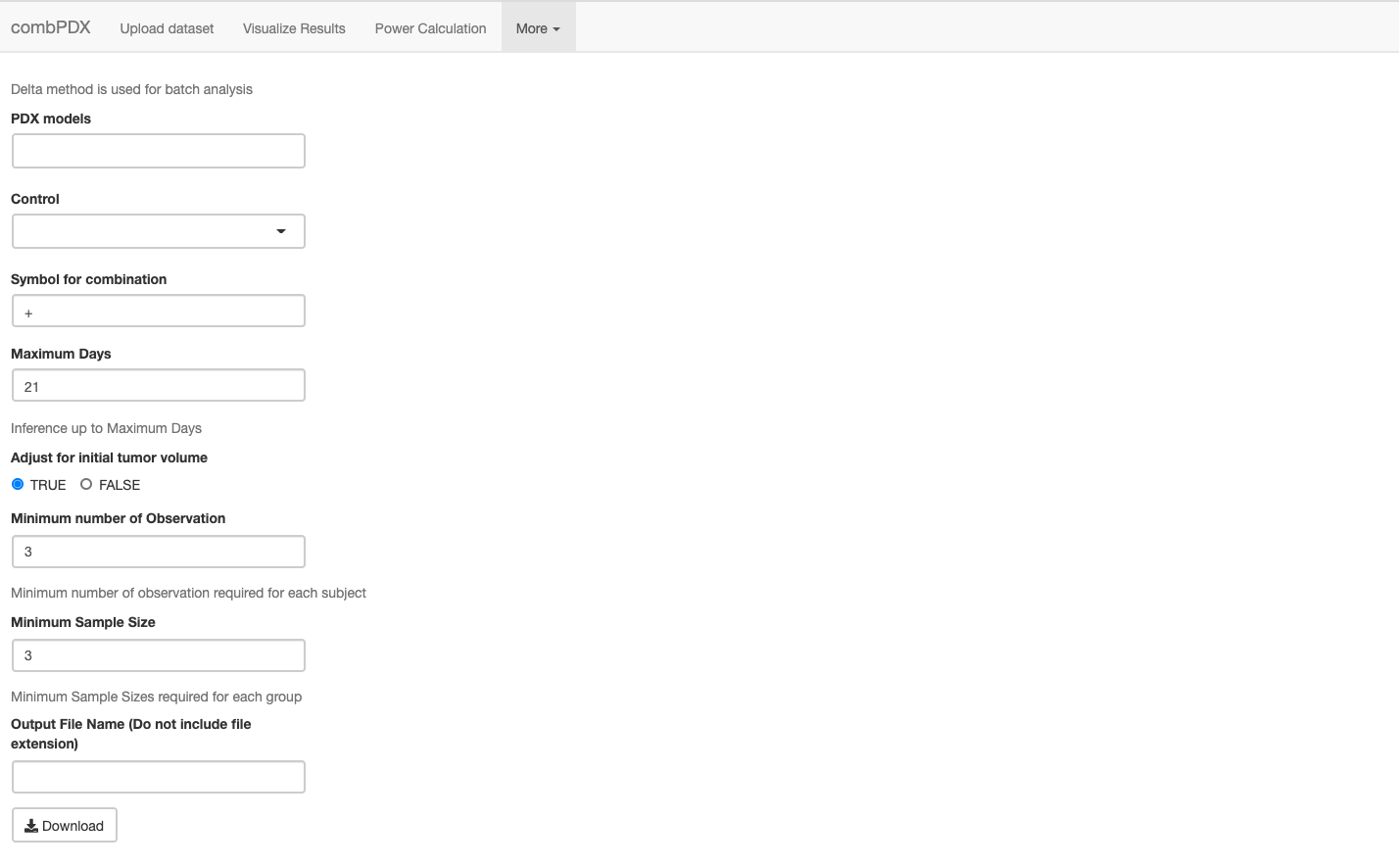


Figure S7: The Batch analysis tab in combPDX conducts Combination Index analysis on multiple combination drugs and provides a R markdown report for all the PDX models.

# **Section S6: Supplementary Tables**

Table S1: Coverage probabilities of the 95% confidence interval for each reference model using the bootstrap interval or Delta method with various sample sizes.

| Evaluated  Model  Data  Generating  Model | | Highest Single Agent | | Response Additive | | Bliss Independence | |
| --- | --- | --- | --- | --- | --- | --- | --- |
|  |  | Delta | Bootstrap | Delta | Bootstrap | Delta | Bootstrap |
|  | N = 5 | | | | | | |
| Highest Single Agent | Model 1 | 0.926 | 0.960 | 0.927 | 0.954 | 0.932 | 0.961 |
| Response Additive |  | 0.915 | 0.961 | 0.913 | 0.959 | 0.934 | 0.962 |
| Bliss Independence |  | 0.907 | 0.945 | 0.914 | 0.955 | 0.933 | 0.955 |
| Highest Single Agent | Model 2 | 0.927 | 0.951 | 0.930 | 0.959 | 0.932 | 0.961 |
| Response Additive |  | 0.910 | 0.943 | 0.906 | 0.960 | 0.934 | 0.962 |
| Bliss Independence |  | 0.904 | 0.940 | 0.919 | 0.954 | 0.933 | 0.955 |
|  | N = 10 | | | | | | |
| Highest Single Agent | Model 1 | 0.933 | 0.947 | 0.925 | 0.945 | 0.940 | 0.948 |
| Response Additive |  | 0.931 | 0.943 | 0.922 | 0.942 | 0.932 | 0.938 |
| Bliss Independence |  | 0.936 | 0.942 | 0.933 | 0.948 | 0.944 | 0.948 |
| Highest Single Agent | Model 2 | 0.936 | 0.947 | 0.936 | 0.946 | 0.940 | 0.948 |
| Response Additive |  | 0.929 | 0.932 | 0.934 | 0.944 | 0.932 | 0.938 |
| Bliss Independence |  | 0.927 | 0.935 | 0.935 | 0.946 | 0.944 | 0.948 |

Table S2. Summary table of treatment information and sample size for PDXs in NSCLC.

| PDX models | Combination Drug (A+B) | Sample Sizes | | | | |
| --- | --- | --- | --- | --- | --- | --- |
|  |  | $n_{c}$ | $n_{A}$ | $n_{B}$ | $n_{\mathrm{AB}}$ | Total |
| TC211 | KRT232+Navitoclax | 5 | 3 | 3 | 3 | 14 |
| TC247 | KRT232+Navitoclax | 7 | 8 | 7 | 8 | 30 |
| TC257 | KRT232+Navitoclax | 5 | 5 | 5 | 5 | 20 |
| TC393 | KRT232+Navitoclax | 3 | 3 | 3 | 3 | 12 |
| TC429 | KRT232+Navitoclax | 11 | 7 | 5 | 6 | 29 |
| TC494 | KRT232+Navitoclax | 11 | 7 | 6 | 7 | 31 |
| TC562 | KRT232+Navitoclax | 3 | 3 | 3 | 3 | 12 |
| TC680 | KRT232+Navitoclax | 10 | 8 | 9 | 10 | 37 |
| TC247x | KRT232+Navitoclax | 3 | 3 | 3 | 4 | 13 |
| TC241 | KRT232+Trametinib | 9 | 3 | 3 | 5 | 20 |
| TC255 | KRT232+Trametinib | 6 | 6 | 5 | 5 | 22 |
| TC314 | KRT232+Trametinib | 6 | 3 | 3 | 6 | 18 |
| TC383 | KRT232+Trametinib | 3 | 3 | 3 | 3 | 12 |
| TC551 | KRT232+Trametinib | 3 | 3 | 3 | 3 | 12 |
| TC616 | KRT232+Trametinib | 3 | 3 | 3 | 5 | 14 |
| TC453 | KRT232+Trametinib | 11 | 5 | 5 | 7 | 28 |
| TC386 | KRT232+Trametinib | 10 | 4 | 4 | 7 | 25 |
| TC429 | KRT232+Trametinib | 11 | 7 | 3 | 6 | 27 |
| TC664 | KRT232+Trametinib | 6 | 3 | 3 | 3 | 15 |
| TC429 | Trametinib+ Navitoclax | 11 | 3 | 5 | 3 | 22 |

Table S3 Summary table of combination indices along with confidence intervals on day 10 and global combination indices under each of the reference model for PDXs in NSCLC.

| PDX models | Treatment (AB) | Combination Index (95% confidence interval) | | | | | |
| --- | --- | --- | --- | --- | --- | --- | --- |
|  |  | HSA day10 | Global HSA | RA day10 | Global RA | BI day10 | Global BI |
| TC211 | KRT232  +  Navitoclax | -0.09  [-0.25- 0.08] | -0.06  [-2.56- 3.34] | -0.18  [-0.34,-0.02] | -0.15  [-0.4- 0.25] | -0.34  [-0.65,-0.03] | -0.28  [-1.04- 0.82] |
| TC247 |  | -0.06  [-0.19- 0.06] | -0.03  [-0.14- 0.12] | -0.13  [-0.33- 0.07] | -0.09  [-0.28- 0.13] | -0.24  [-0.62- 0.13] | -0.17  [-0.51- 0.24] |
| TC257 |  | -0.12  [-0.43- 0.18] | -0.17  [-0.48- 0.56] | -0.24  [-0.5- 0.01] | -0.23  [-0.52- 0.07] | -0.46  [-0.95- 0.02] | -0.45  [-1.01- 0.18] |
| TC393 |  | 0.05  [-0.16- 0.25] | 0.04  [-0.99- 0.77] | -0.06  [-0.23- 0.11] | -0.07  [-0.78- 0.56] | -0.11  [-0.44- 0.22] | -0.11  [-1.23- 0.44] |
| TC429 |  | 0.22  [-0.02- 0.46] | 0.16  [-0.06- 0.41] | -0.02  [-0.15- 0.12] | -0.07  [-0.22- 0.06] | 0.01  [-0.27- 0.3] | -0.08  [-0.37- 0.18] |
| TC494 |  | 0.01  [-0.14- 0.16] | 0.03  [-0.13- 0.24] | -0.06  [-0.22- 0.11] | -0.06  [-0.22- 0.07] | -0.09  [-0.39- 0.2] | -0.09  [-0.36- 0.17] |
| TC562 |  | 0.07  [-0.02- 0.16] | 0.03  [-0.09- 0.29] | 0.03  [-0.07- 0.14] | -0.08  [-0.49- 0.21] | 0.07  [-0.13- 0.27] | -0.12  [-0.76- 0.26] |
| TC680 |  | 0.05  [-0.17- 0.26] | 0.05  [-0.2- 0.24] | -0.02  [-0.17- 0.14] | -0.03  [-0.2- 0.1] | -0.02  [-0.33- 0.29] | -0.05  [-0.38- 0.23] |
| TC247x |  | 0.23  [0.01- 0.45] | 0.2  [-0.01- 0.99] | 0  [-0.14- 0.14] | -0.02  [-0.23- 0.16] | 0.06  [-0.23- 0.35] | 0.04  [-0.28- 0.42] |
| TC241 | KRT232  +  Trametinib | 0.24  [-0.11- 0.59] | 0.19  [-0.19- 0.72] | -0.05  [-0.27- 0.17] | -0.05  [-0.31- 0.16] | -0.01  [-0.44- 0.43] | 0  [-0.46- 0.45] |
| TC255 |  | 0.15  [-0.2- 0.5] | 0.14  [-0.39- 0.55] | -0.1  [-0.34- 0.14] | -0.12  [-0.41- 0.1] | -0.14  [-0.63- 0.36] | -0.18  [-0.8- 0.31] |
| TC314 |  | -0.13  [-0.48- 0.22] | -0.08  [-0.49- 0.79] | -0.4  [-0.71- -0.1] | -0.29  [-0.75- 0.07] | -0.75  [-1.3, -0.17] | -0.52  [-1.39- 0.4] |
| TC383 |  | 0.23  [0.04- 0.42] | 0.09  [-0.25- 0.57] | 0.05  [-0.14- 0.24] | 0  [-0.22- 0.2] | 0.13  [-0.25- 0.51] | 0.02  [-0.41- 0.44] |
| TC551 |  | -0.16  [-0.5- 0.19] | -0.18  [-1.93- 0.47] | -0.05  [-0.55- 0.46] | -0.03  [-0.49- 0.57] | -0.14  [-0.97- 0.7] | -0.15  [-0.82- 0.69] |
| TC616 |  | -0.58  [-0.77,-0.38] | -0.44  [-0.66,-0.12] | -0.34  [-0.79- 0.1] | -0.26  [-0.78- 0.04] | -0.74  [-1.55- 0.07] | -0.53  [-1.6- 0.07] |
| TC453 |  | 0.18  [-0.11- 0.48] | 0.17  [-0.06- 0.41] | -0.12  [-0.28- 0.05] | -0.11  [-0.29- 0.03] | -0.16  [-0.5- 0.18] | -0.13  [-0.48- 0.15] |
| TC386 |  | 0.12  [-0.15- 0.4] | 0.11  [-0.18- 0.43] | -0.03  [-0.3- 0.24] | -0.05  [-0.57- 0.23] | -0.01  [-0.5- 0.49] | -0.03  [-0.81- 0.46] |
| TC429 |  | 0.2  [-0.08- 0.48] | 0.16  [-0.19- 0.44] | -0.04  [-0.19- 0.11] | -0.07  [-0.22- 0.06] | -0.01  [-0.33- 0.31] | -0.05  [-0.39- 0.21] |
| TC664 |  | 0.07  [-0.36- 0.5] | 0.02  [-1.09- 1] | -0.35  [-0.63,-0.07] | -0.36  [-0.71,-0.05] | -0.49  [-1.05- 0.06] | -0.51  [-1.28- 0.09] |
| TC429 | Trametinib  +  Navitoclax | 0.53  [0.31- 0.75] | 0.3  [-0.03- 0.61] | 0.06  [-0.08- 0.21] | -0.02  [-0.17- 0.12] | 0.32  [0.05- 0.59] | 0.1  [-0.17- 0.37] |

Table S4: the required variables and their description in the input data for combPDX.

| Variable | Description | Format |
| --- | --- | --- |
| Models | The experiments ID that corresponding to A, B, C, and AB treatment groups | Character |
| Mice ID | Unique Identification for each mouse in the experiments | Character |
| Treatment | Treatment information for each mouse | Character |
| Volume | Tumor Volume | Numeric |
| DaysPostT0 or Date | Days post first observation or date when the tumor volume is measured | Numeric or Date (m/d/Y) |

Table S5: example data format for the input data for combPDX.

| Model | MiceID | Treatment | Volume | Date |
| --- | --- | --- | --- | --- |
| PDX1 | M1 | A | 224.7 | 2/18/2021 |
| PDX1 | M1 | A | 260 | 2/22/2021 |
| PDX1 | M1 | A | 321 | 2/27/2021 |
| PDX1 | M2 | C | 240.3 | 2/18/2021 |
| PDX1 | M2 | C | 300.4 | 2/22/2021 |

**References**

Berenbaum, M. C. (1989), "What is synergy?," Pharmacol Rev, 41 (2), 93-141.

Bliss, C. I. (1939), "The toxicity of poisons applied jointly 1," Annals of applied biology, 26 (3), 585-615.

Foucquier, J., and Guedj, M. (2015), "Analysis of drug combinations: current methodological landscape," Pharmacol Res Perspect, 3 (3), e00149.

Geary, N. (2013), "Understanding synergy," Am J Physiol Endocrinol Metab, 304 (3), E237-253.

Greco, W. R., Bravo, G., and Parsons, J. C. (1995), "The search for synergy: a critical review from a response surface perspective," Pharmacol Rev, 47 (2), 331-385.

Houghton, P. J., Morton, C. L., Tucker, C., Payne, D., Favours, E., Cole, C., Gorlick, R., Kolb, E. A., Zhang, W., Lock, R., Carol, H., Tajbakhsh, M., Reynolds, C. P., Maris, J. M., Courtright, J., Keir, S. T., Friedman, H. S., Stopford, C., Zeidner, J., Wu, J., Liu, T., Billups, C. A., Khan, J., Ansher, S., Zhang, J., and Smith, M. A. (2007), "The pediatric preclinical testing program: description of models and early testing results," Pediatr Blood Cancer, 49 (7), 928-940.

Lehár, J., Zimmermann, G. R., Krueger, A. S., Molnar, R. A., Ledell, J. T., Heilbut, A. M., Short, G. F., 3rd, Giusti, L. C., Nolan, G. P., Magid, O. A., Lee, M. S., Borisy, A. A., Stockwell, B. R., and Keith, C. T. (2007), "Chemical combination effects predict connectivity in biological systems," Mol Syst Biol, 3, 80.

Mer, A. S., Ba-Alawi, W., Smirnov, P., Wang, Y. X., Brew, B., Ortmann, J., Tsao, M.-S., Cescon, D. W., Goldenberg, A., and Haibe-Kains, B. (2019), "Integrative Pharmacogenomics Analysis of Patient-Derived Xenografts," Cancer Research, 79 (17), 4539-4550.

Ortmann, J., Rampášek, L., Tai, E., Mer, A. S., Shi, R., Stewart, E. L., Mascaux, C., Fares, A., Pham, N.-A., Beri, G., Eeles, C., Tkachuk, D., Ho, C., Sakashita, S., Weiss, J., Jiang, X., Liu, G., Cescon, D. W., O’Brien, C., Guo, S., Tsao, M.-S., Haibe-Kains, B., and Goldenberg, A. (2020), "KuLGaP: A Selective Measure for Assessing Therapy Response in Patient-Derived Xenografts," bioRxiv, 2020.2009.2008.287573.

Ribba, B., Holford, N., Magni, P., Trocóniz, I., Gueorguieva, I., Girard, P., Sarr, C., Elishmereni, M., Kloft, C., and Friberg, L. (2014), "A Review of Mixed-Effects Models of Tumor Growth and Effects of Anticancer Drug Treatment Used in Population Analysis," CPT: Pharmacometrics & Systems Pharmacology, 3 (5), 113.

Slinker, B. K. (1998), "The statistics of synergism," J Mol Cell Cardiol, 30 (4), 723-731.

Winsor, C. P. (1932), "The Gompertz Curve as a Growth Curve," Proceedings of the National Academy of Sciences of the United States of America, 18 (1), 1-8.
